# Targeting the NPL4 Adaptor of p97/VCP Segregase by Disulfiram as an Emerging Cancer Vulnerability Evokes Replication Stress and DNA Damage while Silencing the ATR Pathway

**DOI:** 10.3390/cells9020469

**Published:** 2020-02-18

**Authors:** Dusana Majera, Zdenek Skrott, Katarina Chroma, Joanna Maria Merchut-Maya, Martin Mistrik, Jiri Bartek

**Affiliations:** 1 Laboratory of Genome Integrity, Institute of Molecular and Translational Medicine, Faculty of Medicine and Dentistry, Palacky University, 77 147 Olomouc, Czech Republic; 2 Danish Cancer Society Research Center, 2100 Copenhagen, Denmark; 3 Division of Genome Biology, Department of Medical Biochemistry and Biophysics, Science for Life Laboratory, Karolinska Institute, 171 77 Stockholm, Sweden

**Keywords:** targeted cancer therapy, disulfiram, NPL4, replication stress, DNA damage, BRCA1, BRCA2, ATR pathway

## Abstract

Research on repurposing the old alcohol-aversion drug disulfiram (DSF) for cancer treatment has identified inhibition of NPL4, an adaptor of the p97/VCP segregase essential for turnover of proteins involved in multiple pathways, as an unsuspected cancer cell vulnerability. While we reported that NPL4 is targeted by the anticancer metabolite of DSF, the bis-diethyldithiocarbamate-copper complex (CuET), the exact, apparently multifaceted mechanism(s) through which the CuET-induced aggregation of NPL4 kills cancer cells remains to be fully elucidated. Given the pronounced sensitivity to CuET in tumor cell lines lacking the genome integrity caretaker proteins BRCA1 and BRCA2, here we investigated the impact of NPL4 targeting by CuET on DNA replication dynamics and DNA damage response pathways in human cancer cell models. Our results show that CuET treatment interferes with DNA replication, slows down replication fork progression and causes accumulation of single-stranded DNA (ssDNA). Such a replication stress (RS) scenario is associated with DNA damage, preferentially in the S phase, and activates the homologous recombination (HR) DNA repair pathway. At the same time, we find that cellular responses to the CuET-triggered RS are seriously impaired due to concomitant malfunction of the ATRIP-ATR-CHK1 signaling pathway that reflects an unorthodox checkpoint silencing mode through ATR (Ataxia telangiectasia and Rad3 related) kinase sequestration within the CuET-evoked NPL4 protein aggregates.

## 1. Introduction

Recent advances in understanding of the altered wiring of cancer cell regulatory pathways, and hence vulnerabilities and dependencies of tumor cells have led to discoveries of new molecular targets potentially exploitable in cancer therapy. As the development of a new drug is time-consuming, very expensive, and prone to frequent failure, drug repurposing as a possible alternative approach to cancer treatment is currently undergoing serious consideration [1]. One of the candidate drugs for repurposing in oncology is disulfiram (tetraethylthiuram disulfide, DSF, commercially known as Antabuse), a cheap and well-tolerated generic drug that has been used for decades to treat alcohol dependency. DSF has shown anticancer activity in preclinical models, and multiple clinical trials to treat various types of human malignancies by DSF are currently underway [2]. We have recently published that DSF is metabolized in vivo into the bis-diethyldithiocarbamate-copper complex (CuET), in a process that requires copper ions, and demonstrated that CuET represents the ultimate anticancer metabolite of DSF in-vivo [3]. Furthermore, our nationwide epidemiological study in Denmark yielded results consistent with the emerging anticancer effects of DSF, documenting a lower risk of death from cancer in those cancer patients who were treated by DSF after their cancer diagnosis [3]. Mechanistically, we reported that CuET causes aggregation and thereby immobilization and dysfunction of NPL4, an essential cofactor of the p97/VCP segregase. This otherwise highly mobile protein complex is involved in the regulation of protein turnover upstream of the proteasome, with important roles in a wide range of cellular processes including fundamental pro-survival stress-tolerance pathways [3].

In a follow-up study devoted to target validation and further mechanistic insights into CuET effects [4], we explored the reported exceptional sensitivity to DSF of human cancer cell lines defective in BRCA1 or BRCA2 tumor suppressors, key components of the genome integrity maintenance machinery [4,5]. We found that CuET spontaneously forms from DSF and available copper ions also in cell culture media, and our experiments confirmed NPL4 as the molecular target while excluding the proposed inhibition of aldehyde dehydrogenase (ALDH) [5] and accumulation of toxic acetaldehydes causing DNA-protein and DNA interstrand cross-links [6], as the potential mechanistic explanation for the reported sensitivity of BRCA-defective tumors [4]. In addition to ALDH, we also excluded the proteasome, another previously suggested candidate target of DSF’s anticancer effects, as a valid target. Indeed, we showed that the observed ‘proteasome-inhibition-like features’ triggered by DSF/CuET turned out to be fully attributable to the disabled NPL4 acting upstream of the proteasome [3]. Collectively, these mechanistic studies identified and validated NPL4 as the genuine, and possibly the only or dominant direct molecular target of DSF/CuET responsible for the widely appreciated tumor-inhibitory effects of DSF [3,4]. Indeed, the available evidence in the field now points to CuET-induced aggregation of NPL4 as the key anticancer mechanism of DSF under both in vitro and in vivo conditions, and a promising cancer vulnerability.

Relevant to the present study and the sensitivity of the BRCA-defective cancers to DSF/CuET, we and others previously discovered enhanced replication stress and endogenous DNA damage as a candidate hallmark of cancer [7,8,9,10], thereby pioneering the concept of the ATM-Chk2- and ATR-Chk1-mediated DNA damage response (DDR) checkpoints as important cell-intrinsic barriers against oncogene activation and tumor progression [10,11,12]. Currently, replication stress is recognized to play a prominent role in driving genomic instability and tumorigenesis, while further drug-mediated enhancement of replication stress or inhibition of replication stress-tolerance pathways such as ATR-Chk1 signaling may provide additional targetable vulnerabilities of cancer [13,14]. The main mechanistic consequence of replication stress is the accumulation of ssDNA and stalling of replication forks [15]. The ssDNA stretches become rapidly coated with replication protein A (RPA), thereby facilitating activation of the ATR-Chk1 signaling module and subsequent phosphorylation of hundreds of cellular proteins as substrates of ATR and Chk1 kinases [15,16]. These phosphorylation cascades also involve BRCA1 and BRCA2 and help to stabilize the stalled forks, thereby preventing fork collapse, while in parallel limiting the cellular entry into mitosis by activation of the S-M checkpoint [17]. Under inhibition or genetic deficiency of ATR, stalled replication forks tend to collapse, leading to a generation of DNA double-strand breaks (DSBs), which, if unrepaired or misrepaired, can cause chromosomal instability, severe pathologies or cell death [14,18].

With the above-mentioned knowledge as the starting point, here we examined potential mechanistic links between cancer-associated replication stress, DNA damage checkpoint signaling and the functional impact of DSF/CuET treatment on DNA replication and genome integrity maintenance, searching for possible explanations of the overall sensitivity of tumor cells, and the observed preferential sensitivity of cancer cells lacking BRCA1 and BRCA2, to treatment with DSF/CuET.

## 2. Materials and Methods

### 2.1. Cell Culture 

Human non-small cell lung carcinoma H1299 cells expressing a doxycycline (DOX)-inducible BRCA1 and BRCA2 shRNAs, U2OS, MDA-MB-231, MDA-MB-436, U2OS cells expressing NPL4-GFP, U2OS expressing DOX-inducible MUT-NPL4-GFP [3] and U2OS cells expressing ATR-GFP [19] were cultured and maintained in DMEM (Dulbecco’s Modified Eagle Medium, Lonza, Basel, Switzerland), supplemented with 10% fetal bovine serum (Thermo Fisher Scientific, Waltham, MA, USA) and 1% penicillin/streptomycin (Sigma-Aldrich, St. Louis, MO, USA). CAPAN-1 cells were grown in DMEM medium, supplemented with 20% fetal bovine serum and 1% penicillin/streptomycin. H1299 expressing a DOX-inducible BRCA1 and BRCA2 shRNA were kindly provided [5]. For efficient BRCA1 and BRCA2 knockdown cells were cultivated in the presence of 2 μg/mL DOX for at least three days. 

### 2.2. Immunoblotting

Equal amounts of cell lysates were separated by SDS-PAGE on hand casted gels and then transferred onto the nitrocellulose membrane. The membrane was blocked in Tris-buffered saline containing 5% milk and 0.1% Tween 20 for 1 h at room temperature and then incubated 1 h at room temperature with primary antibodies, followed by detection with secondary antibodies. Secondary antibodies were visualized by ELC detection reagent (Thermo Fisher Scientific, Waltham, MA, USA).

### 2.3. Immunofluorescence

Cells were seeded on plastic inserts in 12-well dishes. The next day, cells were treated with compounds at indicated concentrations and subsequently either pre-extracted (0.1% Triton X 100 in Phosphate-Buffered Saline(PBS) for 2 min or fixed with formaldehyde for 15 min at room temperature, washed with PBS and permeabilized with 0.5% Triton X-100 in PBS for 5 min. After PBS washes, the cells on the plastic inserts were immunostained with primary antibody for 1 h at room temperature, followed by PBS washes and staining with fluorescently-conjugated secondary antibody for 60 min at room temperature. Nuclei were visualized by 4′,6-diamidino-2-phenylindole (DAPI, 1 µg/mL) staining at room temperature for 2 min. For NPL4 staining, the cells were pre-extracted (0.1% Triton X 100 in PBS, for 2 min) and fixed with −20 °C methanol for 15 min at room temperature, washed with PBS and permeabilized with 0.5% Triton X-100 in PBS for 5 min. After PBS washes, the cells on the plastic inserts were immunostained with primary antibody for 120 min at room temperature, followed by PBS washes and staining with fluorescently-conjugated secondary antibody for 60 min at room temperature. Dried plastic inserts with cells were mounted using Vectashield mounting medium (Vector Laboratories, Burlingame, CA, USA), and images were acquired using the Zeiss Axioimager Z.1 platform.

### 2.4. Ethynyldeoxyuridine (EdU) and Bromodeoxyuridine (BrdU) Incorporation and Detection

To detect active DNA replication, cells were incubated with 10 μM EdU (Life Technologies, Carlsbad, CA, USA) for 30 min, fixed, permeabilized and stained using Click-iT reaction (100 mM Tris pH 8.5, 1 mM copper sulfate, 100 μM ascorbic acid, 1 μM azide Alexa fluor 488 (Life Technologies, Carlsbad, CA, USA) for 30 min at room temperature. To detect ssDNA, cells were incubated with 10 μM BrdU (Sigma) for 24 h, then BrdU was washed out, and cells were incubated with the tested compounds as indicated. After pre-extraction and fixation in buffered formol, the incorporated BrdU was detected by an anti-BrdU antibody (BD Biosciences, San Jose, CA, USA) without denaturation.

### 2.5. Image Quantification

Images were acquired using the Olympus IX81 fluorescence microscope and ScanR Acquisition software. The scans were quantified in automated image and data analysis software ScanR Analysis. The data was further analyzed in the STATISTICA 13 software tool (Dell Software, Round Rock, TX, USA).

### 2.6. DNA Combing

H1299 cells were treated with 125 nM CuET for 5 h and subsequently pulsed with 5-Iodo-2’-deoxyuridine (IdU, 20 μM) for 30 min, washed and pulsed with 5-Chloro-2’-deoxyuridine (CIdU, 200 μM) for additional 30 min. DNA replication was stopped by ice-cold PBS. Cells were collected and embedded in 0,5% insert agarose plugs. The plugs were incubated for 32 h in buffer containing proteinase K at 50 °C. Plugs were then washed with TE buffer and melted at 68 °C. The obtained solution was further digested overnight with Agarase I at 42 °C. The next day, the concentration of DNA was measured on nanodrop and combed on silanized cover glasses (Matsunami, Japan) with a speed of 0,3 mm/s. The cover glasses with combed DNA were baked at 60 °C, dehydrated with 70%, 90%, and 100% ethanol series for 3 min each. DNA was denatured at 75 °C in 2xSSC, 50% formamide for 2 min. Next, the cover glasses were dehydrated with a 70%, 90% and 100% ice-cold ethanol series for 5 min each, air dried, blocked using 1% BSA in PBS-Tween for 1 h at 37 °C and subsequently incubated with primary antibodies, mouse anti-BrdU for IdU detection (1:5) and rat anti-BrdU for CIdU detection (1:25) for 1 h at 37 °C. After several washes with PBS-Tween, cover glasses were incubated with secondary antibodies goat anti-mouse A488 (1:100) and goat anti-rat A549 (1:100) for 30 min at 37 °C. After several washes with PBS-Tween, cover glasses were air-dried, mounted, and images of DNA fibers were acquired using the Zeiss Axioimager Z.1 platform.

### 2.7. Estimation of DNA Replication Origin Density 

After the treatment by tested compounds, cells were pulsed with EdU (10 μM) for 20 min, then harvested and resuspended in cold PBS (1 million of cells per 1 mL). 2 μL of cell suspension was applied on glass slides (Superfrost Plus, Thermo Fisher) and allowed to partially evaporate for 5 min, then mixed with a lysis buffer (50 mM EDTA and 0.5% SDS in 200 mM Tris-HCl, pH 7.5) and incubated for 2 min. Slides were tilted to 15° to allow the spreading of fibers. After drying, the samples were fixed in methanol/acetic acid solution for 15 min and thoroughly washed. EdU was detected by click reaction using Alexafluor 488 azide. The signal was further enhanced by anti-Alexa fluor 488 antibody (A-11094, Thermo Fisher) and secondary antibody. DNA was visualized by YOYO-1 (Molecular Probes) staining (1 μM for 20 min). Fiber images were acquired using the Zeiss Axioimager Z.1 platform, and the number of DNA replication origins was calculated on single well-stretched DNA fibers. A conversion factor of 2.59 kb/μm was used in calculations [20]. 

### 2.8. Cell Fractionation for Triton X Insoluble Pellets

Cells were treated as indicated, washed in cold PBS and lysed in lysis buffer (50 mM Tris-HCl, pH 7.5, 150 mM NaCl, 2 mM MgCl2, 10% glycerol, 0.5% Triton-X100, protease inhibitor cocktail by Roche) for 2 min, under gentle agitation at 4 °C. Then, cells were scraped to Eppendorf tubes and kept for another 10 min on ice with vortex steps. Next, the lysate was centrifuged at 20,000× *g* for 10 min at 4 °C. Insoluble fraction and supernatant were re-suspended in Laemmli Sample Buffer (1X final concentration; 10% glycerol, 60 mM Tris-HCl, pH 6.8, 2% SDS, 0.01% bromophenol blue, 50 mM dithiothreitol).

### 2.9. Laser Micro-Irradiation

U2OS cells stably expressing GFP-ATR were seeded into 24-well plates with a glass-bottom (Cellvis) 24 h before laser micro-irradiation in a density of 6 × 105 cells/mL. After seeding the cells into the 24 well plates, the specimen was first placed on an equilibrated bench for 20 min at room temperature (RT) to ensure equal cell distribution and then placed into an incubator. CuET was added to cells 5 h before micro-irradiation in final concentrations of 250 nM and 500 nM. Twenty minutes before laser micro-irradiation, cells were pre-sensitized towards UV-A wavelength by 20 µM 8-Methoxypsoralen (8-MOP) and placed inside Zeiss Axioimager Z.1 inverted microscope combined with the LSM 780 confocal module. Laser micro-irradiation was performed at 37 °C via X 40 water immersion objective (Zeiss C-Apo 403/1.2WDICIII), using a 355 nm 65 mW laser set on 100% power to induce the DNA damage. The total laser dose that can be further manipulated by the number of irradiation cycles was empirically set to two irradiation cycles. Subsequent immunofluorescence detection and quantitative analysis of the striation pattern in photo-manipulated samples were essentially performed as described previously [21].

### 2.10. Antibodies and Chemicals 

The following antibodies were used for immunoblotting: BRCA1 antibody (Santa Cruz Biotechnology, Dallas, TX, USA, D-9), rabbit polyclonal antibody against BRCA2 (Bethyl, Montgomery, TX, USA, A300-005A) antibody and mouse monoclonal antibody against β-actin (Santa Cruz Biotechnology, C4), lamin B (Santa Cruz Biotechnology, sc-6217), α-Tubulin (Santa Cruz Biotechnology, sc-5286), anti-ubiquitin lys48-specific (Merck Millipore, Burlington, MA, USA, clone Apu2) Chk1 (Santa Cruz, Biotechnology, sc-8404), phospho-Chk1 S317 (Cell Signalling, Danvers, MA, USA, 2344), phospho-Chk1 S345 (Cell Signalling, 2348), RPA (Abcam, ab16855, Cambridge, UK), phospho-RPA S33 (Bethyl, A300-246A), ATR (Santa Cruz Biotechnology, N-19). For immunofluorescence were used the following antibodies: γH2AX (Merck Millipore, 05-636), cyclin A (Santa Cruz Biotechnology, H-3, Santa Cruz Biotechnology, sc-239), RPA (Abcam, ab16855), Rad51 (Abcam, ab63801), NPL4 (Santa Cruz Biotechnology, D-1), p97 (Abcam, ab11433), ATR (Santa Cruz Biotechnology, N-19). For DNA combing assay following antibodies were used: anti-BrdU (BD Biosciences, Franklin Lakes, NJ, USA, BD 347580) and rat anti-BrdU (Abcam ab6323).

Chemicals used in this study were as follows: CuET (bis-diethyldithiocarbamate-copper complex, TCI chemicals), disulfiram (Sigma, St. Louis, MO, USA), bortezomib (Velcade, Janssen-Cilag International N.V.), bathocuproinedisulfonic acid (Sigma, St. Louis, MO, USA), CB-5083 (Selleckchem, Houston, TX, USA), hydroxyurea (Sigma, St. Louis, MO, USA), AZD6738 (AstraZeneca, London, UK).

### 2.11. Field Inversion Gel Electrophoresis (FIGE)

Treated cells, as indicated in the main text, were trypsinized and melted into 1.0% InCert-Agarose inserts. Subsequently, agarose inserts were digested in a mixture of 10 mM Tris-HCl pH 7.5, 50 mM EDTA, 1% N-laurylsarcosyl, and proteinase K (2 mg/mL) at 50 °C for 24 hr and washed five times in Tris-EDTA (TE buffer, 10 mM Tris-HCl pH 8.0, 100 mM EDTA). The inserts were loaded onto a separation gel 1.0% agarose mixed with GelRed^®^ solution (10,000x). Run conditions for the DNA fragments separation were: 110 V, 7.5 V/cm, 16 h, forward pulse 11 s, reverse pulse 5 s in 1X Tris-acetic acid-EDTA (TAE buffer 40 mM Tris, 20 mM acetic acid, 1 mM EDTA).

### 2.12. Alkaline Comet Assay

The alkaline comet assay was performed essentially as described in [22]. Briefly, CAPAN-1 and MDA-MB-436 cells were treated with 250 nM CuET or 2 mM hydroxyurea (HU) for 5 h, collected and resuspended in PBS (7500 cells/µL). Cells (75000) were then mixed with 37 °C low melting point agarose (Lonza, Basel, Switzerland), spotted on the normal melting point agarose (Invitrogen, Waltham, MA, USA) pre-coated slides and left to sit for 10 min at 4 °C. Slides were then immersed in the cold alkaline lysis buffer for 2 h at 4 °C. Slides were washed three times with the cold alkaline electrophoresis buffer and electrophoresis was performed (25 min, 4 °C, 0.6 V/cm). Slides were then washed with cold PBS and ddH_2_O, dehydrated in cold graded ethanol, air-dried and stored at room temperature. For staining, slides were rehydrated with ddH_2_O, stained with Sybr Gold (1:4000 in TE buffer; Thermo Fisher Scientific, Waltham, MA, USA), washed with PBS and mounted with Mowiol (Sigma-Aldrich, St. Louis, MO, USA). Images were acquired using a fluorescent microscope (Carl Zeiss, Oberkochen, Germany), a 20x air immersion objective (Carl Zeiss, Oberkochen, Germany) and Comet Assay IV software (Perceptive Instruments, Haverhill, UK). Presented results are from the technical duplicate. Alkaline lysis buffer: 1.2 M NaCl, 100 mM Na_2_EDTA, 0.1% sodium lauryl sarcosinate, 0.26 M NaOH (pH > 13, 4 °C, prepared fresh); alkaline electrophoresis buffer: 0.03 M NaOH, 2 mM Na_2_EDTA (pH 12.3, 4 °C).

## 3. Results

### 3.1. CuET Causes DNA Damage Preferentially Detectable in S/G2-Phase Cells

**Figure 1:**
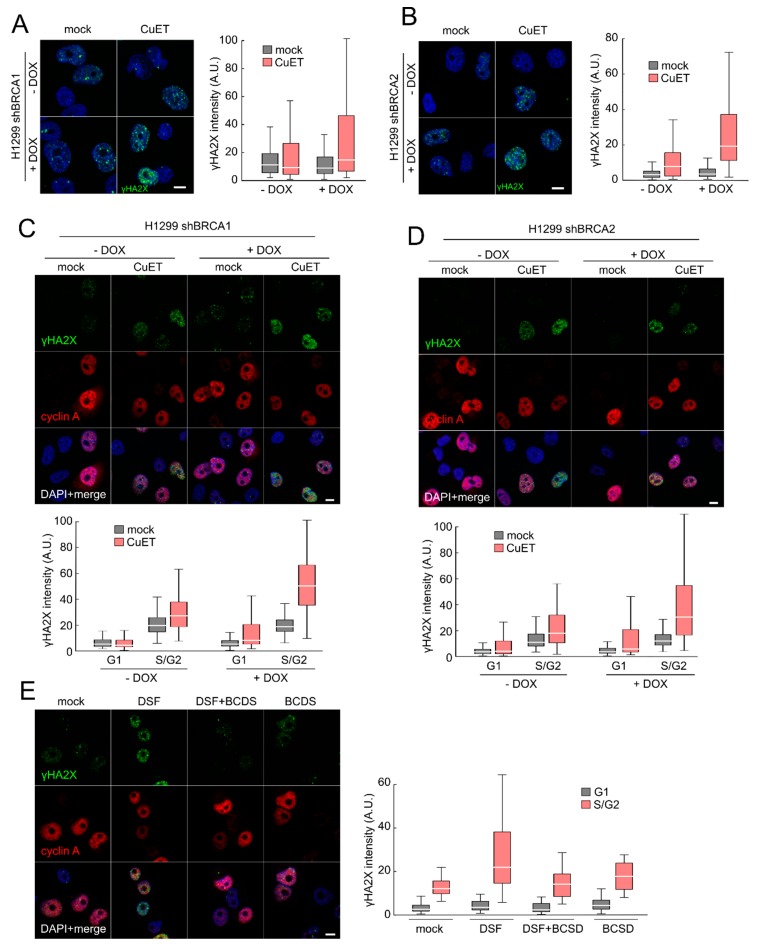
Disulfiram’s metabolite bis-diethyldithiocarbamate-copper complex (CuET) causes DNA damage preferentially in S/G2 cells deficient for BRCA1 or BRCA2 proteins. H1299 cells expressing (doxycycline-) DOX-inducible shBRCA1 (**A**) or shBRCA2 (**B**) were cultivated for at least three days in DOX-containing media and then treated with CuET (250 nM) for 5 h, and γH2AX intensity was analyzed by quantitative microscopy. (**C**) H1229 shBRCA1 cells or (**D**) H1299 shBRCA2 cells were treated as in (A) a γH2AX intensity was quantified with respect to cyclin A positivity defining S/G2 phase. (**E**) H1229 shBRCA2 cells pre-incubated with DOX were treated with disulfram (DSF) (500 nM), bathocuproinedisulfonic acid (BCDS) (50 μM), or their combination for 5 h and γH2AX intensity was quantified. Box plot represents 25–75 quartiles, median, and whiskers non-outlier range. Scale bars = 10 μm.

To initiate our current study, we first wished to assess the impact of CuET on DNA damage in cultured human cancer cells, including isogenic cell pairs with experimentally altered components of the DDR machinery. To this end, we employed the established H1299 lung cancer model allowing for DOX-inducible shRNA-mediated depletion of BRCA1 or BRCA2 [4,5]. Indeed, treatment of these cell lines with CuET resulted in an increased formation of γH2AX foci as well as enhanced overall γH2AX signal intensity, established surrogate markers for chromatin response to DSBs and overall DNA damage signaling by the upstream DDR kinases, respectively (Figure 1A,B; Supplementary Figure S1A,B). Notably, the CuET-evoked increase of γH2AX was more pronounced in the BRCA1- and BRCA2-depleted cells compared with their BRCA-proficient counterpart H1299 cells (unexposed to DOX) (Figure 1A,B; Supplementary Figure S1B). To clarify whether such DNA damage could also be caused by DSF itself, we treated the BRCA2-depleted H1299 cells with DSF in cell culture settings where the cells were first pre-treated by the copper chelator bathocuproinedisulfonic acid (BCDS), a manipulation that we previously reported prevents the otherwise spontaneous and rapid formation of CuET from DSF and copper in cell culture media [4]. As expected, when used alone, DSF caused a similar increase in DNA damage formation as CuET, however when DSF was combined with the copper chelator BCDS pre-treatment step, the γH2AX-inducing effect of DSF was completely abrogated (Figure 1E). These results showed that the DNA damage observed after the treatment with DSF depends on the copper-dependent spontaneous formation of CuET in the culture media, thereby establishing that analogous to the anticancer effects, the active DNA damage-inducing compound is the CuET metabolite, rather than DSF itself.

Next, we pursued our observation that the increase of γH2AX was apparent only in a subset of cells in a given exponentially growing cell population, suggesting that the DNA damage could be cell cycle-dependent. To examine this possibility, we again treated the above mentioned H1299 cells with CuET, yet in the subsequent immunofluorescence analysis, we double stained the cells for γH2AX and cyclin A, an approach commonly used to distinguish cells in G1 phase (cyclin A negative) from those in S/G2 phases (cyclin A positive). Notably, the CuET-induced γH2AX was preferentially seen in cyclin A positive cells, and this cell-cycle effect was even more pronounced in the BRCA1- and BRCA2-depleted cells (Figure 1C,D, Supplementary Figure S1C). The preference of elevated γH2AX intensity in cyclin A positive cells was also confirmed in additional human cancer cell lines (Supplementary Figure S1D,E), thereby excluding a possibility that such genotoxic effects of CuET could be restricted to the H1299 cell model. 

Overall, we conclude from these results that the DNA damage-inducing effects of DSF are attributable to its CuET metabolite, include both elevated γH2AX foci formation and overall γH2AX signal intensity, and occur preferentially in cells traversing S/G2 phases.

### 3.2. CuET Treatment Decreases DNA Replication Fork Velocity and Increases the Number of Active Replication Origins

**Figure 2:**
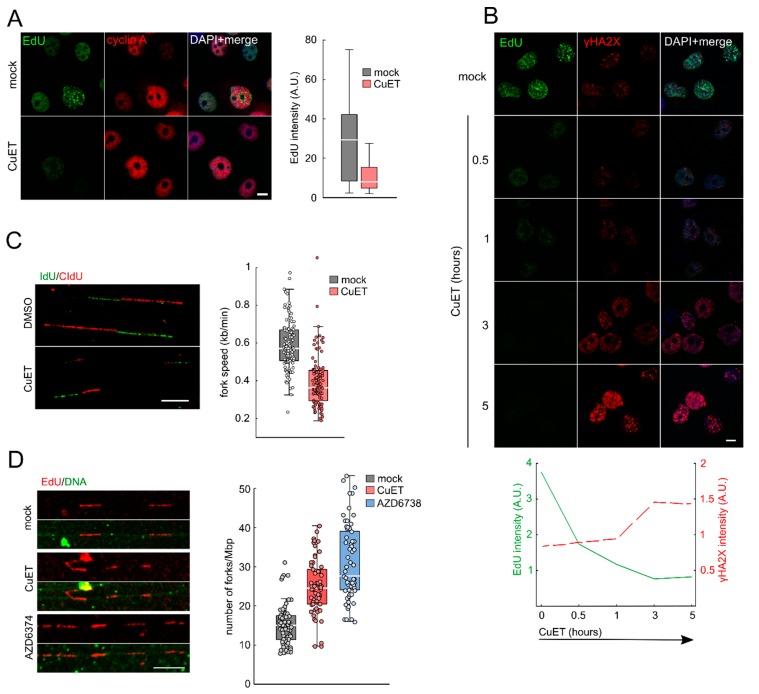
CuET impairs DNA replication. (**A**) H1299 cells were treated with CuET (250 nM) for 3 h, and ethynyldeoxyuridine (EdU) intensity was analyzed in cells positive for cyclin A. (**B**) H1299 cells were treated with CuET (250 nM) for different time points, and EdU and γH2AX intensities were quantified. (**C**) H1299 cells were treated with CuET (125 nM) for 5 h, then pulse-labeled with5-Iodo-2’-deoxyuridine (IdU) and 5-Chloro-2’-deoxyuridine (CIdU) and processed for DNA combing. (**D**) H1299 cells were treated with CuET (250 nM) or AZD6372 (10 μM) for 3 h and then pulsed with EdU and processed for DNA fiber assay. Box plot represents 25–75 quartiles, median, and whiskers non-outlier range. Scale bars = 10 μm.

Since the CuET-induced DNA damage was more apparent in S/G2 cells, we argued that CuET might preferentially interfere with DNA replication. To examine this possibility, we pre-treated H1299 cells with CuET, followed by a pulse-treatment with the thymine analog EdU that becomes incorporated into newly synthesized DNA, allowing visualization of the rate of ongoing DNA replication using fluorescence readouts. Using this approach, we could indeed confirm severe impairment of DNA replication in CuET treated cells, manifested as a decreased EdU signal in H1299 cells (Figure 2A) and also other cell lines, such as human breast cancer MDA-MB-231 and osteosarcoma U2OS cells (Supplementary Figure S2A,B). DNA replication can be halted by the presence of DNA damage [23] and vice versa; replication interference can be the source of DNA damage [13,14]. To address what is the cause and consequence in this scenario, we performed a kinetic study showing that the decrease of EdU incorporation is an early event, preceding the γH2AX foci formation (Figure 2B). This result indicated that the observed DNA damage most likely results from the CuET-induced impairment of DNA replication. To gain more detailed insights into the observed replication interference phenomenon, we employed DNA combing as an assay enabling us to directly assess the effect of CuET on DNA replication fork velocity. H1299 cells were first pre-treated with a rather low concentration of CuET and then pulsed with IdU and CIdU thymine analogs to detect actively replicating DNA, the length of which can be evaluated by fluorescence microscopy-based measurements [24]. Our analysis of the obtained DNA fibers revealed a robust reduction of DNA replication fork velocity after CuET treatment (Figure 2C). Since such decreased DNA replication fork speed is known to trigger firing of dormant replication origins, we next tested the density of active origins using an established DNA fiber assay [22,25]. We quantified the number of origins per 1 Mb of DNA. Indeed, CuET treatment increased the number of active origins compared to untreated cells, similarly to treatment with the ATR kinase inhibitor AZD6738 (Figure 2D), a known activator of latent replication origin firing used here as a positive control [26].

We interpret these results as documenting a previously unsuspected negative impact of CuET on DNA replication, slowing down the fork velocity and concurrently leading to the firing of more dormant origins.

### 3.3. CuET-Induced Replication Stress Leads to DNA Damage that Triggers Homologous Recombination Repair Pathway

**Figure 3:**
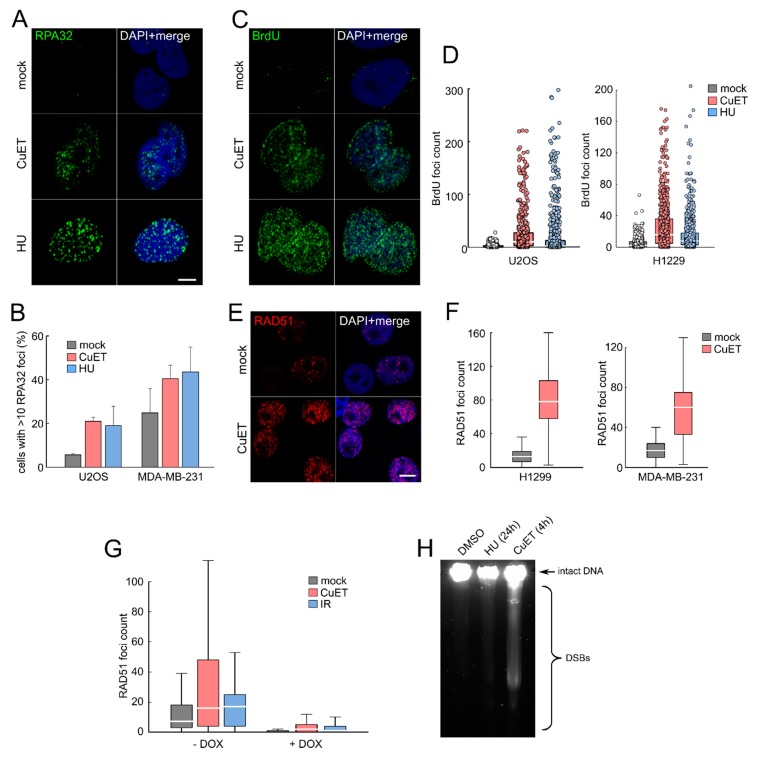
CuET induces replication stress. (**A**) RPA32 foci detection in pre-extracted U2OS cells treated with CuET (250 nM) or hydroxyurea (HU, 2 mM) for 5 h. (**B**) Quantification of cells with more than 10 RPA32 foci treated as in (A) (mean, SD from three independent experiments). (**C**) Formation of single-stranded DNA (ssDNA) visualized by BrdU detected under non-denaturating conditions in U2OS cells treated by CuET (250 nM) and HU (2 mM) for 5 h. (**D**) Quantification of bromodeoxyuridine (BrdU) foci in U2OS and H1299 cells treated as in C. (**E**) Detection of RAD51 foci in pre-extracted H1299 cells treated by CuET (250 nM) for 5 h. (**F**) Quantification of RAD51 foci in cyclin A positive H1299 and MDA-MB-231 cells treated by CuET (250 nM) for 5 h. (**G**) Quantification of Rad51 foci in BRCA2 proficient and deficient H1299 cells after 5-h treatment with 250 nM CuET or 4 Gray (Gy) irradiation. (**H**) FIGE analysis of DSBs in H1299 cells exposed to CuET or HU.Box plot represents 25–75 quartiles, median, and whiskers non-outlier range. Scale bars = 10 μm.

As replication stress is associated with accumulation of ssDNA stretches detectable by RPA32 protein foci or by staining for DNA-incorporated BrdU under non-denaturating conditions [18,27,28], we next assessed these parameters in human cells treated with CuET. Consistent with the CuET-impaired replication forks (see above), we found enhanced RPA32 foci in several cancer cell lines treated with CuET (Figure 3A,B) and also detected incorporated BrdU under non-denaturing conditions (Figure 3C,D). These data suggest that in CuET-treated cells, DNA helicase becomes uncoupled from DNA polymerases, generating stretches of ssDNA in a manner broadly analogous with effects of the replication stress-inducing drugs such as hydroxyurea or aphidicolin [28]. The RPA-coated ssDNA is known to recruit and activate the ATRIP-ATR-CHK1 signaling pathway [29] to stabilize the stalled replication structures, thereby avoiding fork collapse and formation of DSBs [30]. Importantly, these DNA lesions typically require repair by the homologous recombination (HR) repair pathway that encompasses, among other factors, also BRCA1 and BRCA2, the latter being critical for loading of the Rad51 HR repair protein [31,32,33]. To test whether Rad51 is involved in the repair process of lesions caused by the CuET treatment, we stained the cells for Rad51 and searched for the typical DNA-associated Rad51 foci that form within the DSB-flanking chromatin regions under ongoing DNA repair. Indeed, in multiple tested cell lines, the CuET treatment increased the number of Rad51 foci (Figure 3E,F) except for the BRCA2-depleted cells, which are principally incapable of loading Rad51 both after CuET treatment and gamma-irradiation (here used as a positive control) (Figure 3G). The presence of DNA breaks in CuET treated cells was confirmed also by direct physical methods including Field Inversion Gel Electrophoresis (FIGE, detecting largely DSBs) (Figure 3H, Supplementary Figure S3D) and comet assay (Supplementary 3A,B,C, detecting a mixture of single-stranded and double stranded DNA breaks) in BRCA-deficient human cell lines derived from carcinomas of the breast (MDA-MB-436), lung (the H1299 series) and pancreas (CAPAN1), the latter reported by us previously as very sensitive to CuET treatment [3].

Collectively, these results are consistent with CuET inducing replication stress-associated DNA damage that requires HR repair, including Rad51, a process that is defective in the absence of BRCA1 and BRCA2. Consequently, such DNA damage cannot be properly processed in cells lacking the BRCA factors, which explains the higher amount of DNA damage that contributes to the preferential sensitivity of BRCA-deficient cells to DSF [5] and CuET [4].

### 3.4. The ATR Signaling Pathway is Compromised in CuET-Treated Cells

**Figure 4:**
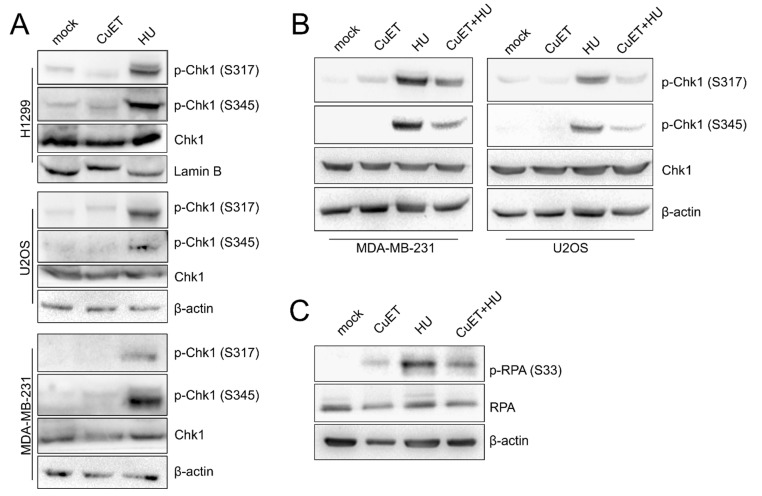
ATR signaling is compromised by CuET. (**A**) Western blotanalysis of phosphorylated forms of Chk1 in various cell lines treated by CuET (250 nM) or HU (2 mM) for 5 h. (**B**) WB analysis of Chk1 phosphorylation in U2OS and MDA-MB-231 cells pre-treated by dimethylsulfoxide (DMSO, mock) or CuET (250 nM) for 2 h and then exposed to HU (2 mM) for additional 3 h. (**C**) WB detection of RPA32 phosphorylation in U2OS cells treated as in B.

In the context of the results obtained so far, we were intrigued by the fact that CuET treatment resulted in DNA breaks relatively quickly within 3–4 h. However, stalled or slowed replication forks should be rather stable for many more hours before turning into DSBs as reported in the U2OS cell line after HU treatment [31] (see also Supplementary Figure S3D). As the prominent role in the stabilization and protection of the stalled forks reflects the function of the RPA-ATRIP-ATR-Chk1 signaling pathway [29,30], we performed immunoblot analysis of extracts from various cell lines treated with CuET, to assess the status of the ATR signaling. In contrast to HU treatment which was used as a positive control, the RPA-ATRIP-ATR-Chk1 signaling pathway was not activated in response to CuET, as manifested by the absence of the ATR-mediated phosphorylations of the effector kinase Chk1: Chk1 S317 and Chk1 S345 (Figure 4A). This result was rather surprising as ssDNA is obviously present in the CuET treated cells (see Figure 3A–D) and also coated by the upstream factor RPA, thereby setting the initial stage for ATR activation and phosphorylation of ATR targets including Chk1. To further investigate whether CuET indeed impairs the RPA-ATRIP-ATR-CHk1 signaling, we treated cells with CuET in the presence of HU. While treatment with HU alone efficiently induced phosphorylation of Chk1 S317 and Ckh1 S345, as expected, the combined treatment with CuET and HU revealed the lack of such Chk1 phosphorylations again, indicating that CuET exerted a dominant effect in suppressing the ATR pathway activity (Figure 4B). These unexpected results were then corroborated by the lack of Serine 33 phosphorylation of yet another ATR substrate, the replication stress marker RPA32, an event seen in the HU-treated control but not in CuET- or combined CuET- and HU-treated cells (Figure 4C). 

Together these results suggest that CuET treatment not only causes replication stress by slowing down and/or stalling replication fork progression but at the same time, it also interferes with the activation of the RPA-ATRIP-ATR-Chk1 signaling cascade that is critical for proper cellular responses to replication stress.

### 3.5. The ATR Signaling Pathway is Compromised in CuET-Treated Cells

**Figure 5:**
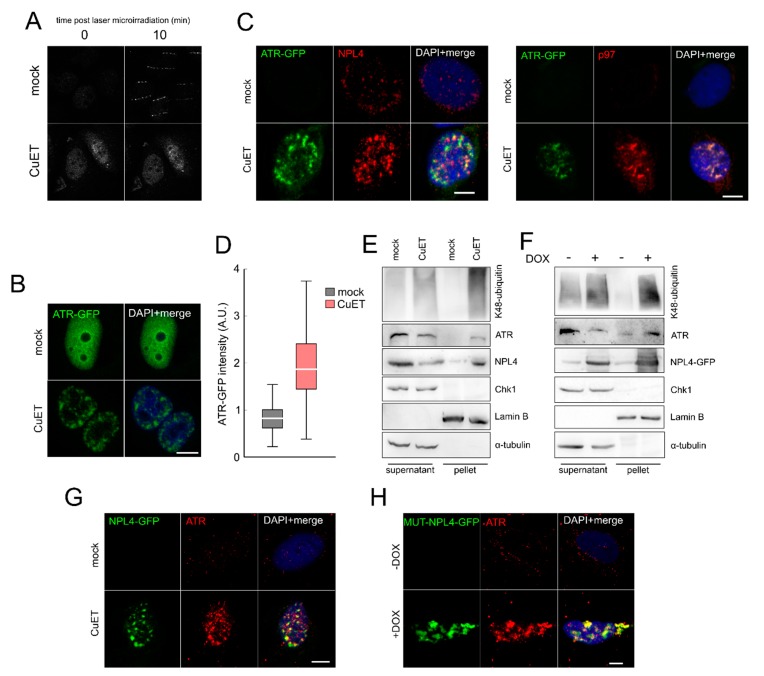
CuET induces immobilization of ATR and its localization to NPL4 aggregates. (**A**) ATR recruitment to sites of damage caused by laser-microirradiation is impaired after CuET treatment (250 nM for 5 h). (**B**) ATR-GFP forms typical nuclear clusters after CuET treatment (250 nM for 5 h). (**C**) Microscopic analysis of co-localization of ATR-GFP with NPL4 and p97 after CuET treatment (250 nM, 3 h) in pre-extracted U2OS cells. (**D**) Quantitative microscopic analysis of pre-extraction resistant ATR-GFP protein in U2OS cells in control and CuET treated cells (250 nM, 5 h). (**E**) WB analysis of immobilized ATR, K48 ubiquitinated proteins, and NPL4 in extracts of CuET-treated (250 nM, 3 h) U2OS cells. (**F**) WB analysis of immobilized ATR, K48 ubiquitinated proteins, and NPL4 in MUT-NPL4-GFP expressing U2OS (Doxycycline induction for 18 h). (**G**) Microscopic analysis of co-localization of NPL4-GFP with ATR after CuET treatment (250 nM, 3 h) in pre-extracted U2OS cells. (**H**) Microscopic analysis of co-localization of MUT-NPL4-GFP with ATR after 18 h doxycycline induction in pre-extracted U2OS cells. Scale bars = 10 μm.

The fact that ATR kinase signaling was suppressed after CuET treatment despite ongoing robust replication stress that also included the formation of ssDNA inspired us to focus directly on the ATR protein and its behavior in response to CuET. As a general readout for analysis of ATR abundance, subcellular localization and function we employed the reporter U2OS cells expressing GFP-labeled ATR (U2OS ATR-GFP) that allowed us to directly assess also recruitment of the ATR protein to acutely inflicted DNA lesions induced by laser microirradiation of psoralen pre-sensitized cell nuclei [19,21]. While in control mock-treated cells, the ATR-GFP protein rapidly formed the expected pattern of fluorescent stripes matching the laser tracks, such recruitment of ATR was markedly impaired after CuET exposure (Figure 5A and Supplementary Figure S4). Moreover, we noticed that in CuET-treated cells without any laser exposure, the otherwise pan-nuclear and generally diffuse ATR-GFP fluorescence signal became altered, forming a pattern that was reminiscent of protein aggregates previously reported by us for the NPL4 protein after CuET treatment [3] (Figure 5B). Indeed, further immunofluorescence analysis confirmed co-localization of ATR-GFP with the NPL4/p97 aggregates formed after CuET treatment (Figure 5C) and general immobilization of the ATR protein was then confirmed by two additional complementary approaches: quantitative microscopy on cultured and pre-extracted U2OS ATR-GFP cells (Figure 5D), and immunoblotting identification of protein translocation from the mobile into the immobile (pre-extraction resistant) protein fraction. Notably, unlike the aggregated immobile ATR protein, the downstream component of the ATR cascade, namely the effector kinase Chk1 was not immobilized after CuET treatment (Figure 5E). To distinguish whether or not ATR immobilization was caused by CuET independently of CuET’s key reported target, the NPL4 protein [3], we employed our U2OS cell model conditionally expressing a mutated form of NPL4-GFP, a protein which tends to aggregate spontaneously when expressed in cells due to the point mutation in the putative zinc-finger domain involved in the interaction with CuET [3]. We have already shown that such spontaneous aggregation of the NPL4-MUT protein mimics multiple aspects of CuET treatment including association and immobilization of various cellular stress-response proteins including HSP70, p97, SUMO, polyUb, and TDP43 with the NPL4 aggregates [3]. Indeed, using this model, we found the association and immobilization of ATR-GFP within the spontaneously formed NPL4-MUT aggregates (Figure 5F,H). 

In summary, these experiments identified NPL4 aggregation, induced by either CuET in the case of wild-type NPL4, or mutation-caused conformational change of the NPL4-MUT protein in the absence of any added CuET, as the primary event and a pre-requisite for the subsequent sequestration of ATR in such NPL4 aggregates, with the ensuing signaling defect of the ATR-Chk1 signaling pathway.

## 4. Discussion

The major advance provided by the results from our present study is the identification of a new mode of cancer cell cytotoxicity evoked by diethyldithiocarbamate-copper complex, CuET [3,4], the anticancer metabolite of the alcohol aversion drug DSF that is currently tested in clinical trials for repurposing in oncology. Indeed, after years of convoluted efforts to understand the tumor-inhibitory effects of DSF, the field has been aided by our discovery of CuET as the ultimate cancer-killing compound that rapidly forms as DSF becomes metabolized under both in vivo [3], and cell culture [4,34] conditions. At the mechanistic level, we found that CuET impairs the cellular protein degradation machinery upstream of the proteasome, by inducing aggregation and immobilization of NPL4, an essential cofactor of the p97/VCP segregase complex [3]. This mechanism helps explain the observed preferential toxicity in cancer cells experiencing high levels of proteotoxic stress, such as multiple myeloma [3]. 

Inspired by the recent intriguing observation that human cancer cells lacking the BRCA1/2 DNA damage response genes are particularly sensitive to DSF [4,5], here we focused on potential genotoxic/replication stress as another aberrant cancer-associated trait [7,8,9,10] that could be triggered and/or enhanced by CuET. Indeed, we have found that CuET induces DNA damage preferentially in S-phase cells consistent with robust impairment of DNA replication, induction of replication stress, and impairment of ATR signaling. The same effects can be recapitulated with replacing CuET by DSF, as the culture media contain traces of copper that enable the spontaneous formation of CuET [34]. We validated the latter notion by combined treatment of cells with DSF and the copper chelator BCDS (Figure 1E), which efficiently precludes the spontaneous formation of CuET [4] and thereby the cellular phenotypes otherwise shared by CuET and DSF. 

The fundamental question that emerges from our present study, and which we address only partially here, is the nature of the precise molecular mechanism behind the CuET-induced replication stress. As CuET impairs the p97/NPL4 pathway that is directly implicated in several processes linked to DNA repair and replication [35], it remains to be seen whether the replication interference could be explained by impacting such processes, including DNA replication, translesion synthesis, DNA-protein crosslinks repair, or termination of replication [36], possibly in a combination. Moreover, p97, together with diverse cofactors, is also directly involved in DSB repair, contributing to the recruitment of the 53BP1 repair factor [37] and also other DDR proteins [38,39,40]. On the other hand, also indirect effects of NPL4 aggregation, for example, the triggered heat-shock response, could plausibly contribute to the phenotypes observed here. In our previous work, we observed that apart from NPL4/p97, the CuET-induced aggregates contain several proteotoxic stress-related proteins, including HSP70, SUMO2/3, polyubiquitin chains, and TDP-43 [3]. Here, we have surprisingly found that also ATR kinase, a key factor required for proper cellular response to replication stress, is trapped and sequestered in the NPL4 aggregates, thus explaining the dysfunction of ATR signaling in CuET-treated cells. Conceptually, given that ATR dysfunction is known to trigger replication stress, a feature we see also after CuET treatment, one could argue that ATR aggregation could represent the primary and/or major cause of the CuET-induced replication stress. On the other hand, our time-course analysis suggests that DNA replication becomes impaired very quickly upon CuET addition, as judged from the EdU staining (Figure 2B), in fact preceding any detectable ATR aggregation. Therefore, we currently believe that the two processes, replication fork stalling, and ATR aggregation are possibly initiated independent of each other and act rather in a complementary manner to cause the observed robust replication stress phenotype. A related emerging question for future work is what brings ATR to the vicinity of the forming NPL4 aggregates in the first place? This issue is speculative at present, and it remains to be seen whether some structural features of ATR, possibly shared by additional proteins, such as unstructured regions or high dependency on chaperones, could be involved. Alternatively, the recruitment to aggregates might share the mechanism of the reported ATR recruitment into areas of high topological stress within the nuclear envelope [41]. ATR might be sequestrated by the aggregates also through direct interaction with NPL4 or due to the global proteotoxic stress-related changes in the cell. The latter scenario would partially resemble the so-called β-sheets-containing protein aggregates that sequester and mislocalize several proteins involved in RNA metabolism and nucleocytoplasmic transport [42]. Alternatively, liquid–liquid phase separation might also be involved in this process. A recent study [43] revealed that acute hyperosmotic stress induces phase separation of the proteasomes and formation of discrete puncta in the nucleus. Interestingly, these structures also contained K48-ubiquitinated proteins or p97 segregase, the proteins also found in NPL4 clusters, raising the question of whether phase separation plays a role in the case of NPL4 aggregation or attraction of other proteins. These questions need to be addressed by dedicated future studies, to help us better understand the effects of NPL4 aggregates on cellular physiology, providing clues about why so many seemingly unrelated phenotypes have so far been described after DSF treatment.

Last but not least, our present results are also highly relevant from the clinical point of view, not least because protein aggregation represents an unorthodox and so far largely unexplored mechanism of action for anticancer drugs. This rather unique mechanism may also contribute to the observed synergistic effects of DSF/copper with either ionizing radiation [44] or the DNA damage-inducing drug temozolomide [45] a combination currently tested in several clinical trials focusing on glioblastoma patients [46,47,48], as well as a combination of DSF with cisplatin [49]. We hope that the data we report here will inspire further research in this rapidly evolving area of biomedicine, and yield additional effective therapies based on combining DSF/copper (CuET) with other currently used DNA damage-related therapeutic modalities.

Overall, based on our present results we suggest that CuET (DSF/copper) evokes and/or exacerbates replication stress in tumor cells while concomitantly precluding the ATR-mediated pro-survival response to such stress, thereby collectively creating a toxic scenario (understandably more severe in BRCA1/2-defective cells) reminiscent of ‘killing two birds with one stone.’

## Supplementary Materials

The following are available online at https://www.mdpi.com/2073-4409/9/2/469/s1, Figure S1: CuET is causing DNA damage preferentially in S/G2 cells. Figure S2.: CuET impairs DNA replication in MDA-MB-231 and U2OS cells. Figure S3: Detection of DNA breaks after CuET treatment. Figure S4: Microscopy-based quantitative analysis of fluorescence signal in cells.
