# Supplementary material for: Targeting the NPL4 Adaptor of p97/VCP Segregase by Disulfiram as an Emerging Cancer Vulnerability Evokes Replication Stress and DNA Damage while Silencing the ATR Pathway
Source: Cells. 2020 Feb 18;9(2):469. doi: 10.3390/cells9020469 (PMC7072750; doi:10.3390/cells9020469)
Supplement: Supplementary file 1 [file cells-09-00469-s001.pdf]

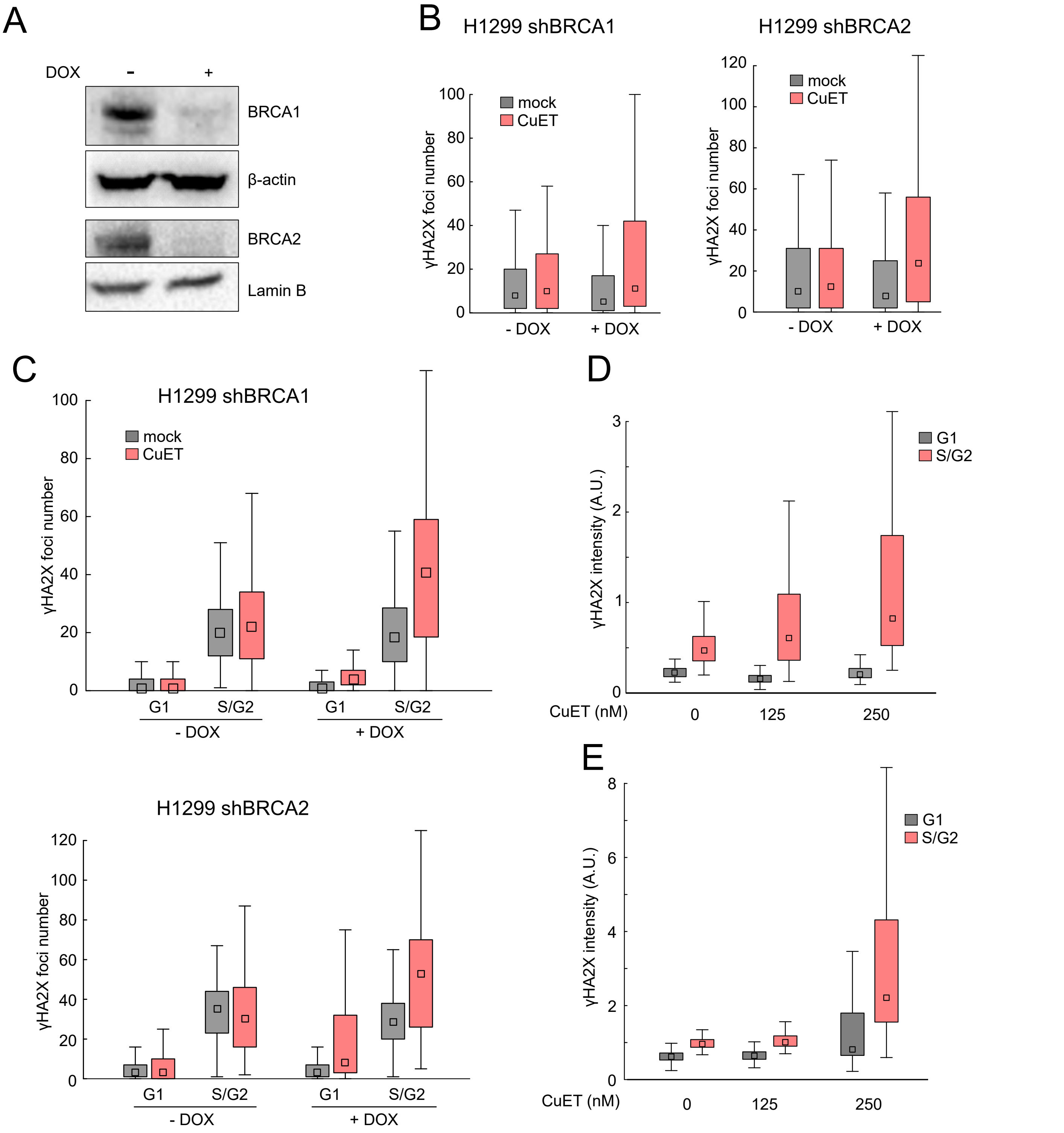


**Supplementary figure 1**: CuET is causing DNA damage preferentially in S/G2 cells. (A) WB analysis of knock-down efficiency in H1299 cells expressing DOX inducible shBRCA1 or shBRCA2 after 3 days of incubation in the medium containing doxycycline. (B) DOX-inducible shBRCA1 or shBRCA2 were cultivated for at least 3 days in DOX-containing media and then treated with CuET (250 nM) for 5 hours and γH2AX foci per nucleus were analysed by quantitative microscopy. (C) H1299 shBRCA2 cells were treated as in (B) a γH2AX foci per nucleus were quantified with respect to cyclin A positivity defining S/G2 phase. (D) MDA-MB-231 and (E) U2OS cells were treated by CuET for 5 hours and γH2AX intensity was quantified with respect to cyclin A positivity defining S/G2 phase.

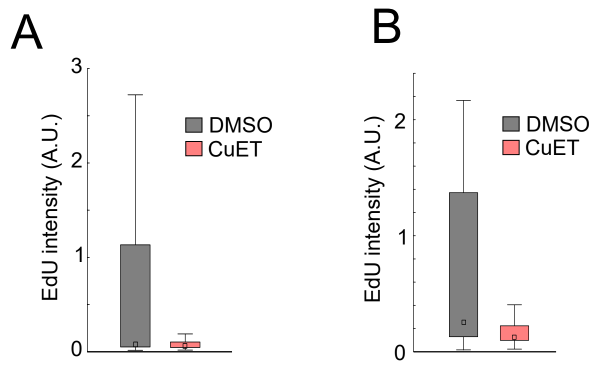


**Supplementary figure 2**: CuET impairs DNA replication in MDA-MB-231 and U2OS cells. (A) MDA-MB-231 and (B) U2OS cells were treated with CuET (250 nM for 5 hours) and EdU intensity was quantified.

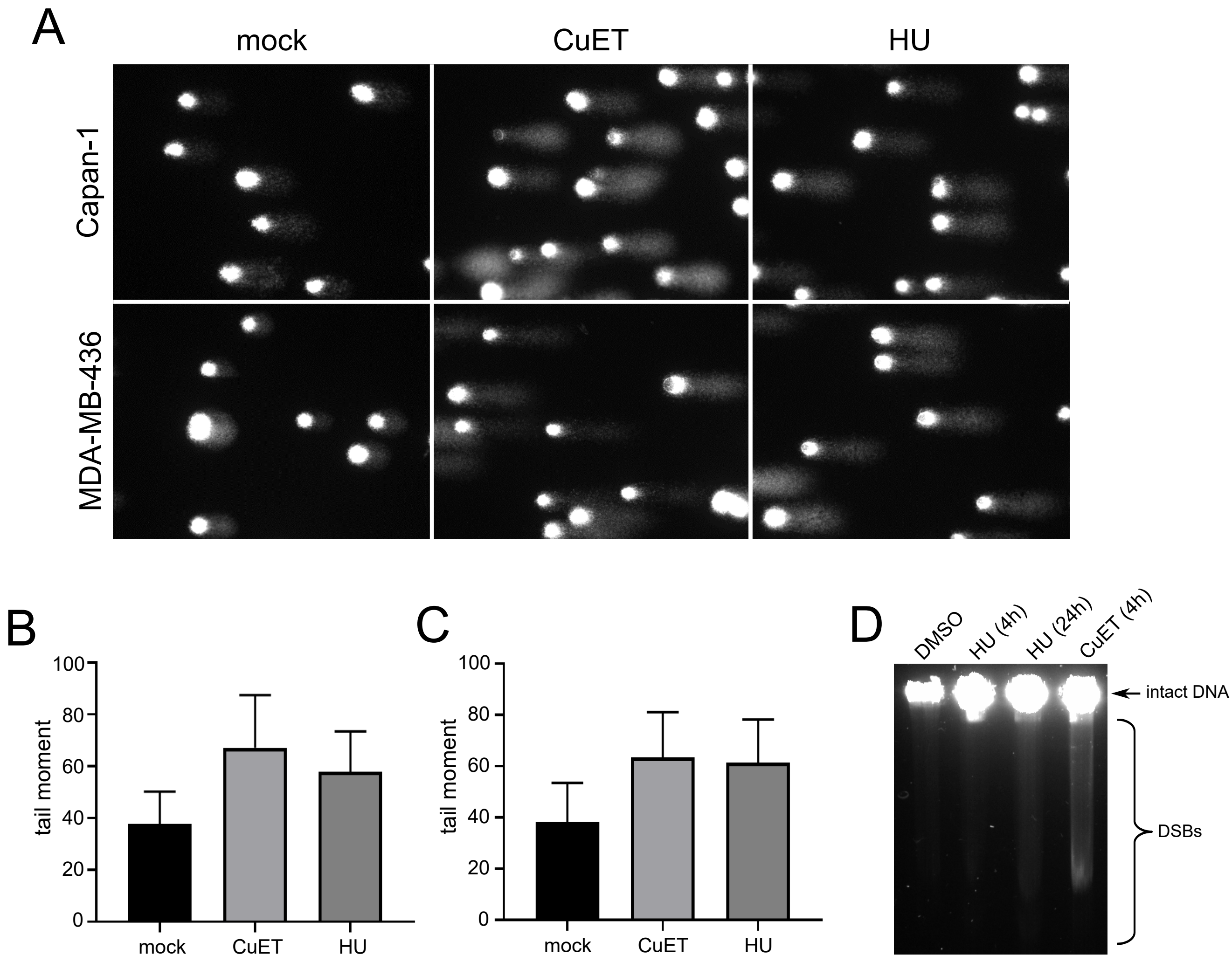


**Supplementary figure 3**: Detection of DNA breaks after CuET treatment. (A) Detection of DNA breaks by alkaline comet assay in Capan-1 and MDA-MB-436 cells treated by CuET (250 nM) or HU (2 mM) for 5 hours. Quantification of tail moment in (B) Capan-1 cells and (C) MDA-MB-436 cells. (D) Detection of DSBs by FIGE in U2OS cells trated by HU (2 mM) or CuET (500 nM).

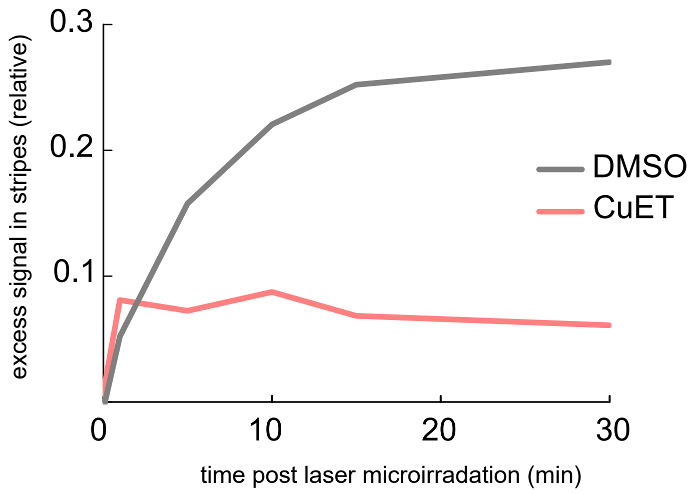


**Supplementary figure 4**: Microscopy-based quantitative analysis of fluorescence signal in cells. Quantification of ATR-GFP excess signal in stripes at different timepoints after laser microirradiation in DMSO and CuET treated cells (250 nM for 5 hours).
